# Companion basil plants prime the tomato wound response through volatile signaling in a mixed planting system

**DOI:** 10.1007/s00299-024-03285-w

**Published:** 2024-07-22

**Authors:** Riichiro Yoshida, Shoma Taguchi, Chihiro Wakita, Shinichiro Serikawa, Hiroyuki Miyaji

**Affiliations:** 1 Laboratory of Horticultural Science, Faculty of Agriculture, Kagoshima University, 1-21-24 Kohrimoto, Kagoshima, 890-0065 Japan; 2 The United Graduate School of Agricultural Sciences, Kagoshima University, 1-21-24 Kohrimoto, Kagoshima, 890-0065 Japan

**Keywords:** Companion plants, Priming, Tomato, Volatile, Wound signaling

## Abstract

**Key message:**

**Volatile compounds released from basil prime the tomato wound response by promoting jasmonic acid, mitogen-activated protein kinase, and reactive oxygen species signaling.**

**Abstract:**

Within mixed planting systems, companion plants can promote growth or enhance stress responses in target plants. However, the mechanisms underlying these effects remain poorly understood. To gain insight into the molecular nature of the effects of companion plants, we investigated the effects of basil plants (*Ocimum basilicum* var. *minimum*) on the wound response in tomato plants (*Solanum lycopersicum* cv. ‘Micro-Tom’) within a mixed planting system under environmentally controlled chamber. The results showed that the expression of *Pin2*, which specifically responds to mechanical wounding, was induced more rapidly and more strongly in the leaves of tomato plants cultivated with companion basil plants. This wound response priming effect was replicated through the exposure of tomato plants to an essential oil (EO) prepared from basil leaves. Tomato leaves pre-exposed to basil EO showed enhanced expression of genes related to jasmonic acid, mitogen-activated protein kinase (MAPK), and reactive oxygen species (ROS) signaling after wounding stress. Basil EO also enhanced ROS accumulation in wounded tomato leaves. The wound response priming effect of basil EO was confirmed in wounded Arabidopsis plants. Loss-of-function analysis of target genes revealed that MAPK genes play pivotal roles in controlling the observed priming effects. *Spodoptera litura* larvae-fed tomato leaves pre-exposed to basil EO showed reduced growth compared with larvae-fed control leaves. Thus, mixed planting with basil may enhance defense priming in both tomato and Arabidopsis plants through the activation of volatile signaling.

**Supplementary Information:**

The online version contains supplementary material available at 10.1007/s00299-024-03285-w.

## Introduction

Agricultural systems worldwide are dominated by industrial approaches that produce single crops under the control of chemical fertilizers and pesticides (Horrigan et al. 2002). However, these systems carry a substantial risk of degrading topsoil, which is required for crop growth, and produce large amounts of greenhouse gasses, which accelerate global warming (Gao et al. 2022). In addition, excessive nitrogen and phosphorus inputs to agricultural lands pollute rivers and lakes, creating severe environmental problems worldwide (Moss 2007). To meet growing agricultural demands while preserving the global environment, there is a need to rapidly establish agricultural practices that conserve the environment. The transportation of agricultural products also emits large amounts of CO_2_, such that shortening the distances between agricultural production and consumption areas (i.e., reducing food mileage) is a critical challenge that requires an aggressive shift from conventional large-scale farming to small-scale farming. For vegetable production, it is also necessary to review subsistence-based production systems in private gardens and urban–suburban production systems. Furthermore, the growing health consciousness among consumers is leading to an expectation of safe and secure agricultural products through reduced chemical pesticide use.

Regenerative agriculture, which aims to restore the natural environment while improving the soil for crop growth, has been proposed to ensure global food security while mitigating these problems (Giller et al. 2021). One step toward regenerative agriculture is the implementation of mixed planting, rather than monocultures. Companion planting, in which compatible crops of different species are grown together, originated in the USA, where indigenous Americans planted a mixture of corn, pumpkins, and beans known as the “three sisters” (Pleasant 2016). Companion planting is generally considered beneficial to plants because of its ability to control pests and diseases (Finch et al. 2003; Parker et al. 2013; George et al. 2013; Fu et al. 2015), optimize soil nutrient supply (Mengel 2001), and improve growing space efficiency (Bomford 2004). However, the specific effects of companion plants remain unclear. A typical example of companion planting is a “push–pull” system, in which natural plant–insect communication is harnessed to reduce herbivory by insects (Pickett et al. 2014). In this system, volatiles released from plants repel or disturb feeding insects while attracting their natural enemies. For instance, volatiles released from companion plants have been reported to effectively protect target plants against aphid or whitefly damage under greenhouse conditions (Ben-Issa et al. 2017a, b; Conboy et al. 2019).

Companion plants can help to enhance the defense systems of a target plant species. For example, volatiles released by mint plants were reported to increase pest resistance in soybean or *Brassica rapa* plants within mixed planting systems (Sukegawa et al. 2018), and volatiles released from injured *Solidago canadensis* were able to inhibit root nodule symbiosis by nitrogen-fixing bacteria on soybean roots (Takahashi et al. 2021). These findings indicate that volatile signaling is strongly involved in the effects of companion planting on target plants. However, few studies have comprehensively investigated this phenomenon, and molecular research is needed to elucidate its underlying mechanism. An understanding of the mechanisms that drive this effect may help to promote the widespread implementation of companion planting for sustainable agriculture and maximal effectiveness within mixed planting systems.

Therefore, the objective of this study was to clarify the molecular basis for the effects of companion planting on target plants in a tomato–basil mixed planting system, primarily focusing on interplant communication. We investigated the effects of mixed plantings on tomato wound response at the gene expression level, with the aim of elucidating the molecular nature of airborne signaling between tomato and basil. To further elucidate this signaling mechanism, we also used Arabidopsis, analyzed its gene loss-of-function mutants, and compared the results with those obtained in tomatoes.

Our results provide scientific evidence that this beneficial effect of companion planting is mainly driven by interplant communication via plant volatile signaling, primarily mediating MAPK and ROS.

## Materials and methods

### Plant materials and growth conditions

Tomato (*Solanum lycopersicum* cv. ‘Micro-Tom’), basil (*Ocimum basilicum* var. *minimum*), and Arabidopsis (Columbia ecotype) were used in this study. All plants were grown in soil consisting of a 1:1 ratio of Metro-Mix (Sun Gro Horticulture, Agawam, MA, USA) to vermiculite within a controlled environmental chamber at 23 °C under a 12-h/12-h light/dark photoperiod. A mixed planting system was established by transplanting germinated tomato and basil seeds into 9-cm-diameter pots.

In a tomato *jai1-1* experiment, homozygous *jai1-1* seedlings were selected from F_2_ populations following the method previously described by Li et al. (2003). Seeds were germinated on a piece of water-saturated filter paper in a closed petri dishes in the dark at 25 °C. After 4–5 days, when the emerging radical was ∼1 cm in length, the filter paper was resaturated with a solution of 1 mM methyl jasmonate (MeJA). Seedlings were grown in the dark for an additional 24–36 h, and selected the seedlings insensitive to MeJA of those did not show reduced root and hypocotyl growth and anthocyanin accumulation in the hypocotyl.

One germinated seed of tomato and basil or tomato and tomato was transplanted into each pot. The distance between plants was 3 cm. After three weeks of growth, the leaves of each plant were wounded with scissors in an area on each side of the leaf, bordering the main leaf vein.

The loss-of-function of Arabidopsis mutants *atmpk3* (SALK_209371) and *atmpk6* (SALK_004221) were obtained from the Arabidopsis Biological Resource Center (ABRC).

### Insect culture and feeding experiments

*Spodoptera litura* (*S. litura*) larvae eclosed from eggs were reared on artificial feed and grown to the second instar stage at 25 °C under a 14-h/10-h light/dark photoperiod. The larvae were carefully put onto the untreated and basil essential oil (EO)-exposed tomato leaves that had excised from seedlings and fed at 23 °C under a 14-h/10-h light/dark photoperiod, then weighed after 3 days to evaluate inhibitory effects on *S. litura* growth. Thirty larvae were included in each treatment.

### Basil EO and volatile compound treatments

Three-week-old tomato and four-week-old Arabidopsis plants grown in 5-cm-diameter pots were placed inside a plant box (7 cm length × 7 cm width × 10 cm height); cotton swabs soaked with basil EO or one of the four tested volatile compounds (linalool, α-terpineol, chavicol, or eugenol) were attached to the bottom of the lid, and the box was closed. After 15 h of exposure, plants were removed from the box and desensitized for 1 h. Then, leaves of each plant were wounded with scissors in one area on each side of the leaf, bordering the main leaf vein. Basil EO was extracted using the hydro distillation method (Tongnuanchan and Benjakul 2014). About 100 g of fresh basil leaves and 200 mL of water are put into a 1000 mL boiling flask and heated using a heating mantle-type heater (200W/100 V) for one hour. After the extraction process, the oil accumulated in the extraction tool was carefully removed and then put into dark-colored vials. Extracted oils were stored at 4 °C until use.

### Quantitative polymerase chain reaction (qPCR) analysis

Total RNA was isolated from tomato and Arabidopsis leaves using TRIzol reagent (Invitrogen, Carlsbad, CA, USA). cDNA was synthesized using ReverTra Ace (Toyobo, Osaka, Japan), in accordance with the manufacturer’s instructions. qPCR was performed using the Eco Real-Time PCR System (Illumina, San Diego, CA, USA) using the KAPA SYBR FAST qPCR kit (Sigma-Aldrich, St. Louis, MO, USA). The qPCR cycling protocol consisted of 40 cycles of 95 °C for 5 s and 60 °C for 30 s. Primer sequences used in this analysis are listed in Table S1. Expression levels for each target gene were normalized to the levels of *ACTIN* (for tomato) and *ACTIN2* (for Arabidopsis). The replication size was *n* = 3 for all experiments.

### ROS determination and quantification

Accumulation of ROS in the leaves was visually detected using 3,3-diaminobenzine (DAB). After the wound stress treatment, tomato or Arabidopsis leaves were incubated with DAB solution (1 mg/mL) at pH 3.8 for at least 12 h. After the incubation with DAB solution, the leaves were put into the 100% ethanol solution to decolorize chlorophyll. H_2_O_2_ accumulation in leaves was quantified using GIMP2 software (Postor et al. 2013). GIMP2 is a free and open-source image editor that is available for GNU/Linux, macOS, Windows, and other operating systems (https://www.gimp.org). The replication size was *n* = 3 to 4 for all experiments.

### Statistical analysis

One-way ANOVA was performed to compare the effects of mix-planting, EO, and each volatile compound. The significance of differences among the treatments was evaluated using a Tukey’s honest significant difference (HSD post hoc test). Differences referred to in the text were statistically significant at *P* < 0.05 unless otherwise stated. Statistical analyses were performed using EZR v.1.60 ((Saitama Medical Center, Jichi Medical University, Saitama, Japan). Results are reported as averages ± standard deviations (SD). A two-sided Student’s *t* test was performed in Fig. 10.

## Results

### Companion planting with basil induced a priming effect on the tomato wound response

**Fig. 1:**
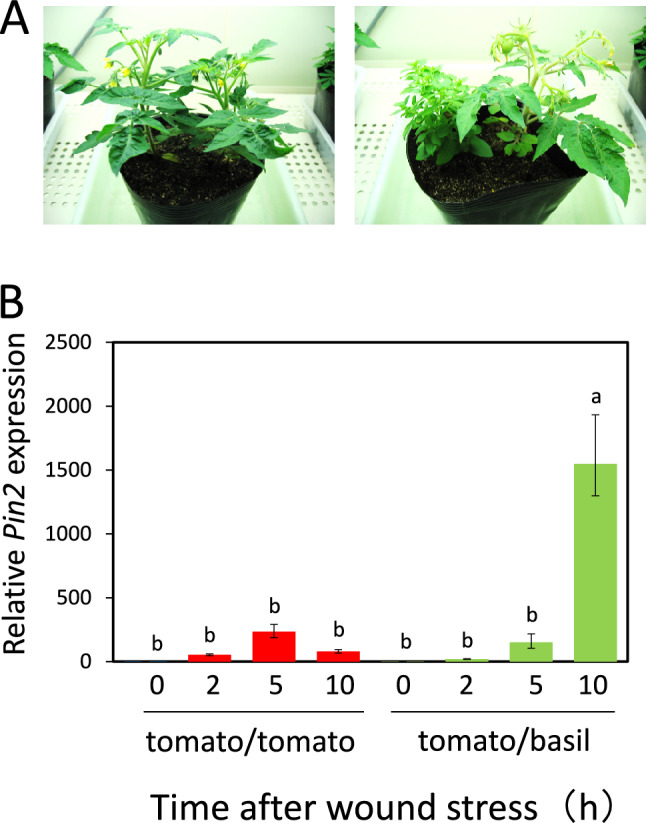
Mixed planting with basil enhanced *Pin2* expression in tomato plants under wounding stress. **a** Tomato plants were grown for 3 weeks with or without basil companion plants. **b** Effects of basil on expression of the wound response gene *Pin2* in tomato leaves. Leaves were wounded on both sides with scissors, sampled at the indicated times, and then subjected to quantitative polymerase chain reaction (qPCR) analysis. Bars represent means ± standard deviations (SDs) from three independent experiments. Different letters indicate significant differences (*P* < 0.05, one-way analysis of variance [ANOVA] followed by Tukey’s test; *n* = 3)

In our experimental system, tomato plants grown with basil were compared to tomato plants grown without basil (Fig. 1a). However, no significant differences, such as plant size, were observed between the two growth conditions (data not shown). To understand the effects of companion basil plants on tomato plants, tomato leaves were subjected to wound stress, followed by analyses of the expression levels of the wound response gene *Pin2* (Farmer and Ryan 1992). The results showed that tomato plants grown with basil rapidly exhibited higher *Pin2* expression levels than tomato plants grown without basil (Fig. 1b).

We performed two independent experiments to determine whether the root system/rhizosphere contributes to the primed wound response (Figs. S1a, S2a). Both experiments were set up to eliminate direct contact between tomato and basil underground. Since both experiments demonstrated the co-cultivation priming effect in wounded tomato leaves (Figs. S1b, S2b), we considered the basil effect to be mainly due to the airborne signal.

### Essential oil (EO) prepared from basil leaves primed the wound response in wounded tomato leaves

**Fig. 2:**
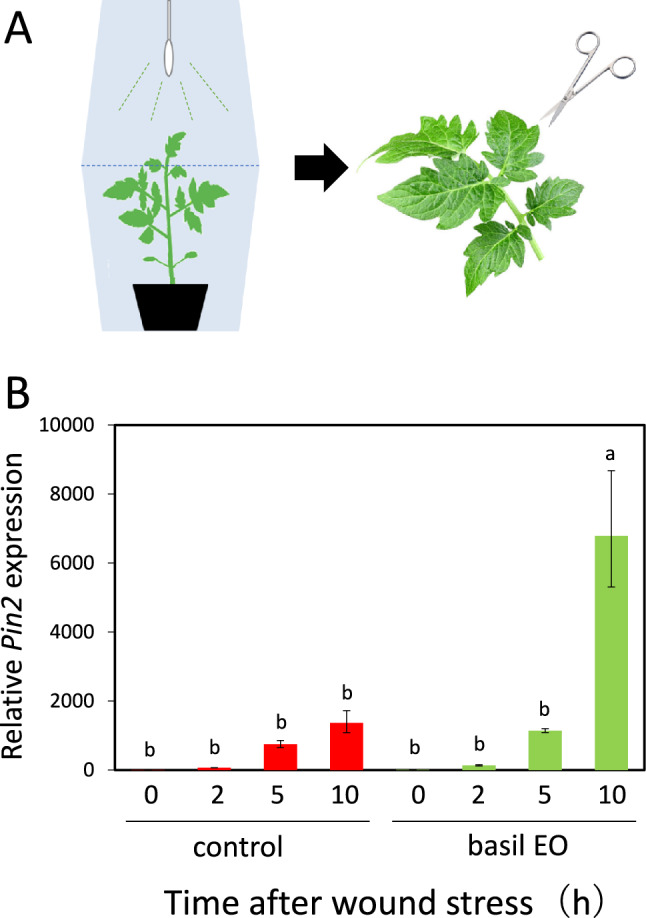
Effects of basil essential oil (EO) on the wounding response in tomato plants. **a** Schematic representation of the experimental setup. Plants were placed inside a box; cotton swabs soaked with 5 mL of basil EO (5 mL of water for controls) were attached to the bottom of the lid, and the box was closed. After 15 h of exposure, the box was opened, and the plant leaves were wounded with scissors after a desensitization period. **b** Effects of basil EO on *Pin2* gene expression in tomato leaves. Tomato leaves were sampled at the indicated times and subjected to qPCR analyses. Bars represent means ± SDs from three independent experiments. Different letters indicate significant differences (*P* < 0.05, one-way ANOVA followed by Tukey’s test; *n* = 3)

To determine whether the observed wound response priming effect was caused by volatiles released from the aboveground parts of basil plants, we exposed tomato plants to purified EO extracted from basil leaves. Tomato plants were placed in plant boxes (Fig. 2a) and exposed to basil EO for 15 h; their leaves were then subjected to wound stress. Next, we examined *Pin2* gene expression in each leaf. Tomato plants that were exposed to basil EO exhibited similar wound response priming to the findings in tomato plants grown with basil plants (Fig. 2b).

### Effects of individual volatile compounds in basil EO on tomato wound response priming

**Fig. 3:**
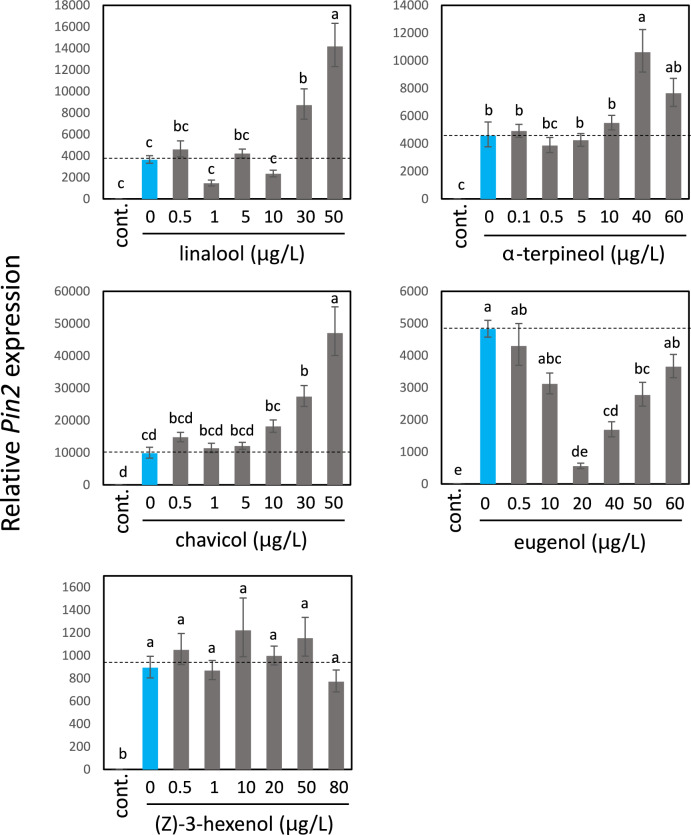
Effects of five volatile compounds on the wound response in tomato plants. Tomato plants were pre-exposed to each compound at the indicated concentrations for 15 h, then wounded with scissors. At 10 h after wounding, tomato leaves were sampled and subjected to qPCR analysis. Bars represent means ± SDs from three independent experiments. Different letters indicate significant differences (*P* < 0.05, one-way ANOVA followed by Tukey’s test; *n* = 3). Leaves were sampled and their *Pin2*-transcript levels were analyzed by qPCR

Next, we investigated which volatile components of the basil EO are involved in the induction of wound response priming. Tomato plants were exposed to four major volatile compounds **(**Politeo et al. 2007) — linalool, α-terpineol, chavicol, and eugenol—at various concentrations (Fig. 2a). Because a previous study showed that (Z)-3-hexenol induced a priming effect in corn seedlings attacked by insects (Engelberth et al. 2004), we included this compound in our experiments. The results showed that linalool, α-terpineol, and chavicol exhibited a priming effect on wound-induced *Pin2* expression compared with the control (Fig. 3), whereas no significant priming effect was observed for eugenol or (Z)-3-hexenol.

### Basil EO strengthened jasmonic acid (JA) signaling in tomato plants

**Fig. 4:**
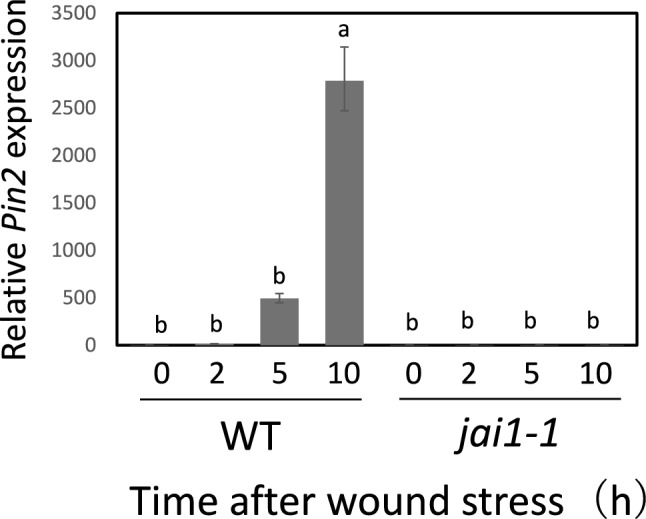
Basil EO promotes jasmonic acid (JA) signaling in tomato plants. Effect of JA-insensitive *jai1-1* mutation on wound-induced *Pin2* expression. Wild-type (WT) and *jai1-1* mutant plants were pre-exposed to basil EO for 15 h, then wounded with scissors. Tomato leaves were sampled at the indicated times, then subjected to qPCR analysis. Bars represent means ± SDs from three independent experiments. Different letters indicate significant differences (*P* < 0.05, one-way ANOVA followed by Tukey’s test; *n* = 3)

**Fig. 5:**
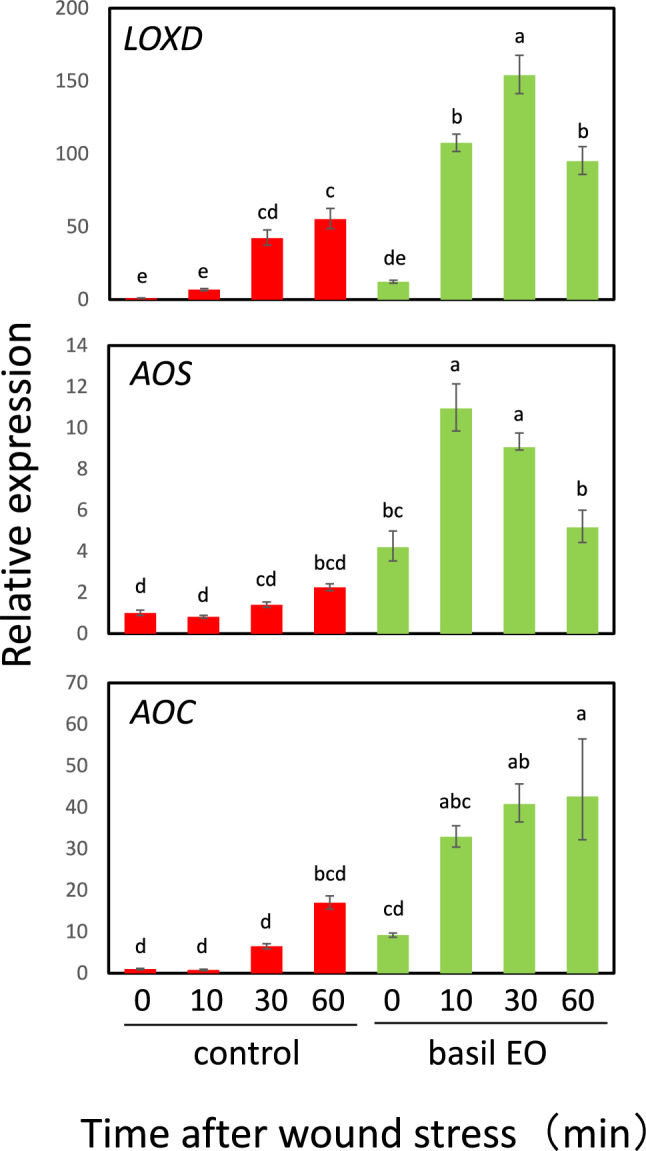
Basil EO enhances wound-induced expression of JA biosynthesis-related genes in tomato plants. Effects of basil EO on the wound-induced expression of JA biosynthesis-related genes *LYPOXYGENASE D* (*LOXD*), *ALLENE OXIDE SYNTHASE* (*AOS*), and *ALLENE OXIDE CYCLASE* (*AOC*). WT plants were pre-exposed to basil EO for 15 h, then wounded with scissors. Tomato leaves were sampled at the indicated times, then subjected to qPCR analysis. Bars represent means ± SDs from three independent experiments. Different letters indicate significant differences (*P* < 0.05, one-way ANOVA followed by Tukey’s test; *n* = 3)

**Fig. 6:**
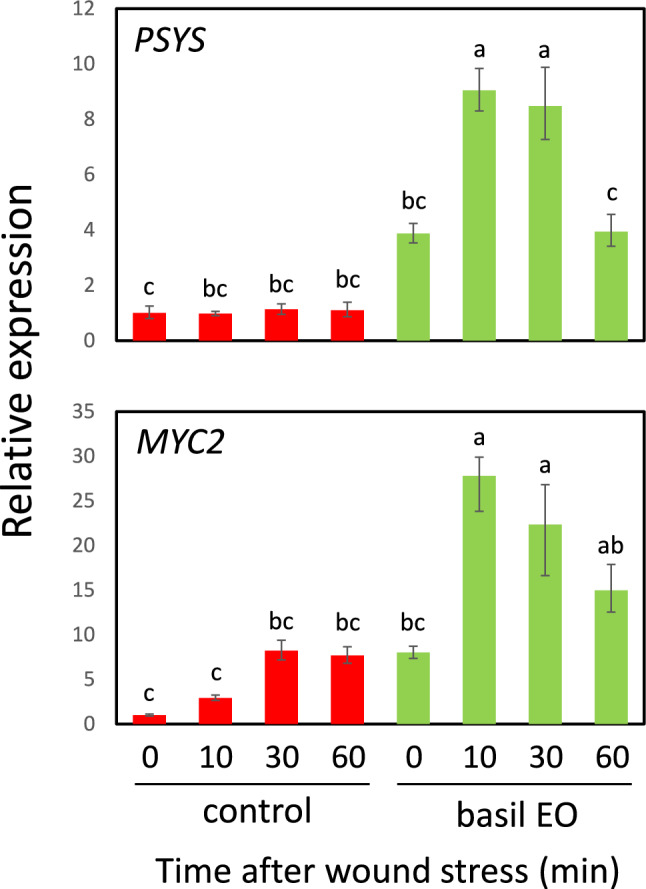
Basil EO enhances wound-induced expression of JA signaling-related genes in tomato plants. Effects of basil EO on the wound-induced expression of JA signaling-related genes *PROSYSTEMIN* (*PSYS*) and *MYC2* in tomato plants. WT plants were pre-exposed to basil EO for 15 h, then wounded with scissors. Tomato leaves were sampled at the indicated times, then subjected to qPCR analysis. Bars represent means ± SDs from three independent experiments. Different letters indicate significant differences (*P* < 0.05, one-way ANOVA followed by Tukey’s test; *n* = 3)

Because tomato *Pin2* gene expression is controlled by JA (Farmer and Ryan 1992), we investigated whether basil EO affects the expression of JA-related genes during wound stress. In tomato plants with a loss-of-function mutation in *JAI1*, the tomato homolog of Arabidopsis *COI1*, we found that *jai1-1* mutants showed strong inhibition of *Pin2* expression after experiencing wound stress (Fig. 4). Next, we examined the effects of basil EO on the induction of JA synthesis genes during the short-term response to leaf wounding. We found that the expression of three essential genes, *LYPOXYGENASE D* (*LOXD*), *ALLENE OXIDE SYNTHASE* (*AOS*), and *ALLENE OXIDE CYCLASE* (*AOC*), were directly and substantially induced by basil EO (Fig. 5). Furthermore, a similar strong priming response was observed for the expression of *MYC2*, a key factor in JA signaling (Boter et al. 2004; Du et al. 2014), as well as *PSY*, a precursor to the plant peptide hormone systemin (Ryan and Pearce 1998) (Fig. 6).

### Basil EO induced the expression of MAPK- and ROS-related genes

**Fig. 7:**
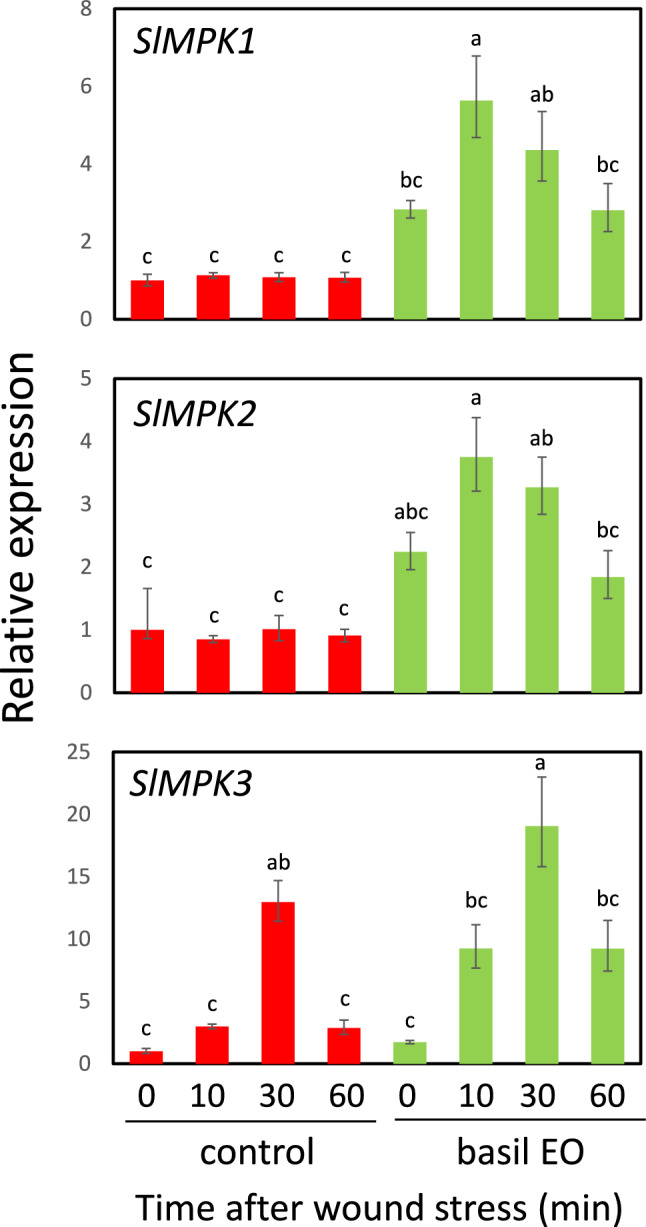
Basil EO induces and promotes the expression of mitogen-activated protein kinase (MAPK) genes in tomato plants. Effects of basil EO on the wound-induced expression of three MAPK genes (*SIMPK1*, *SIMPK2*, and *SIMPK3*) in tomato plants. WT plants were pre-exposed to basil EO for 15 h, then wounded with scissors. Tomato leaves were sampled at the indicated times, then subjected to qPCR analysis. Bars represent means ± SDs from three independent experiments. Different letters indicate significant differences (*P* < 0.05, one-way ANOVA followed by Tukey’s test; *n* = 3)

MAPK is involved in plant-wound signaling and controls endogenous JA levels (Seo et al. 2007). The accumulation of MAPK proteins in cells has been associated with priming induction in plant-stress signaling (Conrath 2011). Therefore, we examined whether basil EO promotes expression of the tomato MAPK genes *SIMPK1*, *SIMPK2*, and *SIMPK3* after wound stress. SlMPK1 and SlMPK2 are the orthologs of Arabidopsis AtMPK6, and are 95% identical at the amino acid level (Stulemeijer et al. 2009). SlMPK3 is the orthologs of Arabidopsis AtMPK3 and is transcriptionally upregulated in response to wounding (Higgins et al. 2007). These MAPKs were shown to function in the systemin-mediated defense response against herbivorous insects (Kandoth et al. 2009). The results of this experiment did not confirm wound-related induction of *SIMPK1* and *SIMPK2* expression in control plants; however, the expression of these genes was significantly induced by pre-exposure to basil EO (Fig. 7). In contrast, *SIMPK3* was transiently expressed after wounding, with a peak at 30 min, and a priming effect was observed in plants pre-exposed to basil EO.

**Fig. 8:**
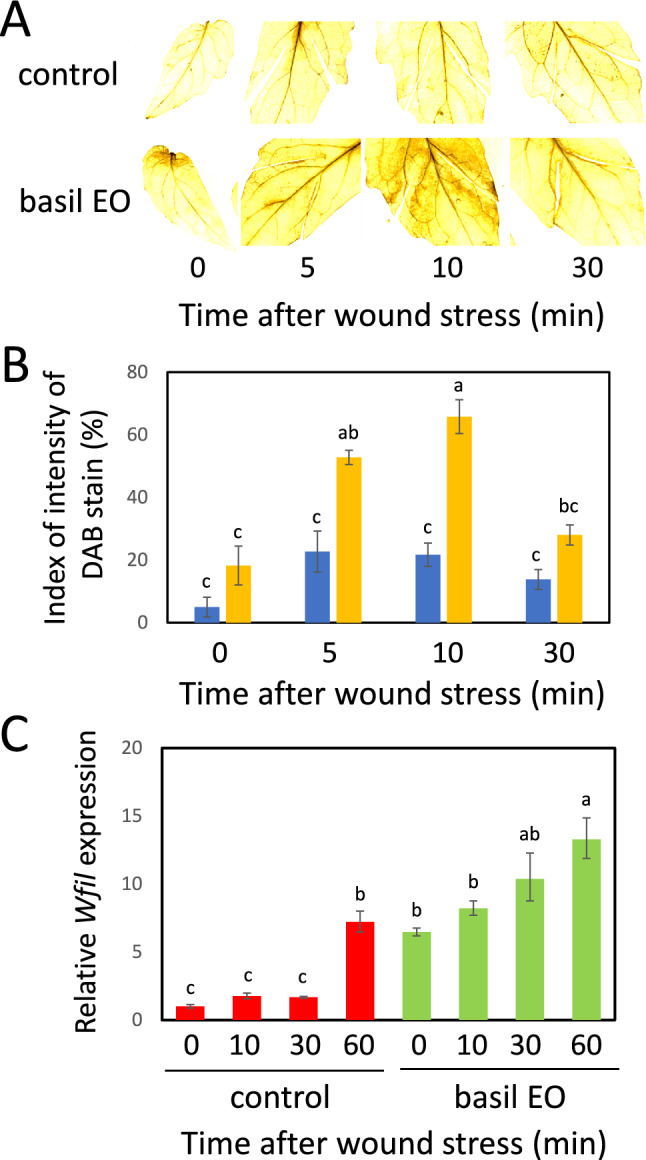
Effects of basil EO on wound-induced reactive oxygen species (ROS) accumulation in tomato leaves. **a** DAB (3,3′-diaminobenzidine) staining of wounded tomato leaves. Tomato plants were pre-exposed to basil EO for 15 h, then wounded with scissors. **b** DAB staining intensity in wounded leaves was quantified using GIMP software. Blue and orange bars represent control and basil EO treatments, respectively. **c** qPCR analysis of transcript levels of the tomato NADPH-oxidase gene *Wfi1* in wounded tomato leaves. Tomato plants were pre-exposed to basil EO for 15 h, then wounded with scissors. Tomato leaves were sampled at the indicated times, then subjected to qPCR analysis. Bars represent means ± SDs from three independent experiments. Different letters indicate significant differences (*P* < 0.05, one-way ANOVA followed by Tukey’s test; *n* = 3)

ROS have also been proposed as strong candidates for controlling priming responses in plants (Pastora et al. 2013). Therefore, we examined the effect of basil EO on ROS accumulation in tomato leaves under wound stress via 3,3′-diaminobenzidine (DAB) staining. The results showed that ROS accumulation was upto threefold higher in basil EO-exposed leaves than in control leaves (Fig. 8a, b). Higher expression levels of *Wfi1*, a key gene for ROS production in tomatoes (Song et al. 2018), were also observed in basil EO-exposed leaves after wounding (Fig. 8c).

### Basil EO promoted the wound response in Arabidopsis leaves

**Fig. 9:**
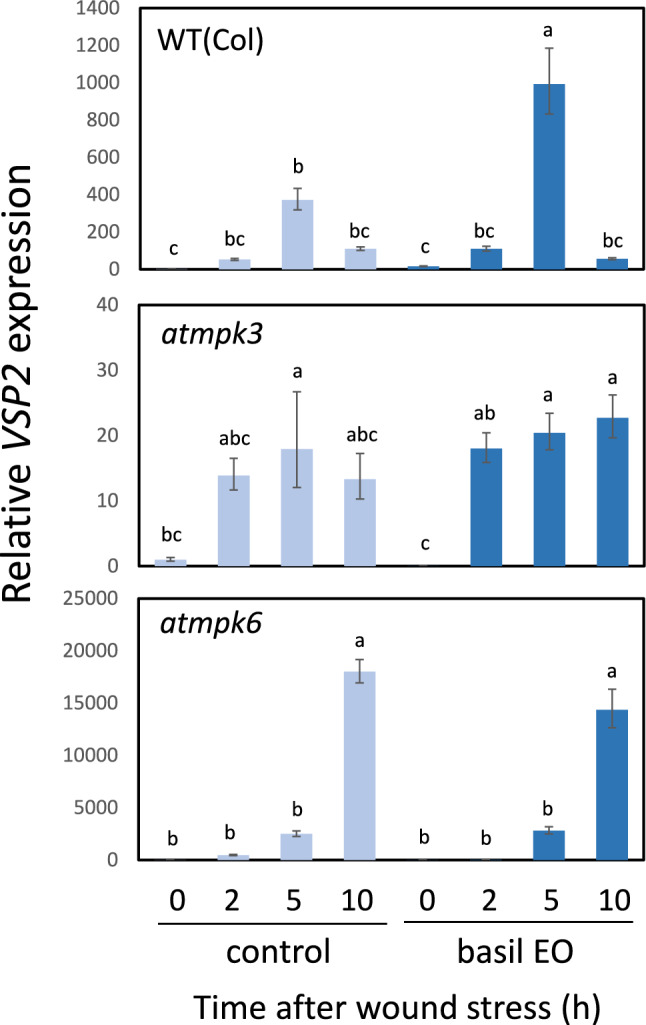
Basil EO promotes the wound response in Arabidopsis through a mechanism mediated by MAPK genes. Effects of basil EO on expression of the wound response gene *VSP2* in Arabidopsis. WT, *atmpk3*, and *atmpk6* plants were pre-exposed to basil EO for 15 h, then wounded with scissors. Arabidopsis leaves were sampled at the indicated times, then subjected to qPCR analysis. Bars represent means ± SDs from three independent experiments. Different letters indicate significant differences (*P* < 0.05, one-way ANOVA followed by Tukey’s test; n = 3)

Because basil EO primed the tomato wound response, we investigated whether a similar effect could occur in Arabidopsis. We exposed Arabidopsis plants to basil EO and subjected them to wound stress. In Arabidopsis leaves exposed to basil EO, we observed enhanced expression of the wound response gene *VSP2* (Fig. 9). We also found that loss-of-function mutations affecting the Arabidopsis MAPK genes *AtMPK3* and *AtMPK6* eliminated the priming effect of basil EO on the wound response (Fig. 9). Furthermore, basil EO did not appear to enhance ROS accumulation in wounded leaves of *atmpk3* or *atmpk6* plants (Fig. S3).

### *S. litura* larvae-fed basil EO-exposed tomato leaves exhibited growth inhibition

**Fig. 10:**
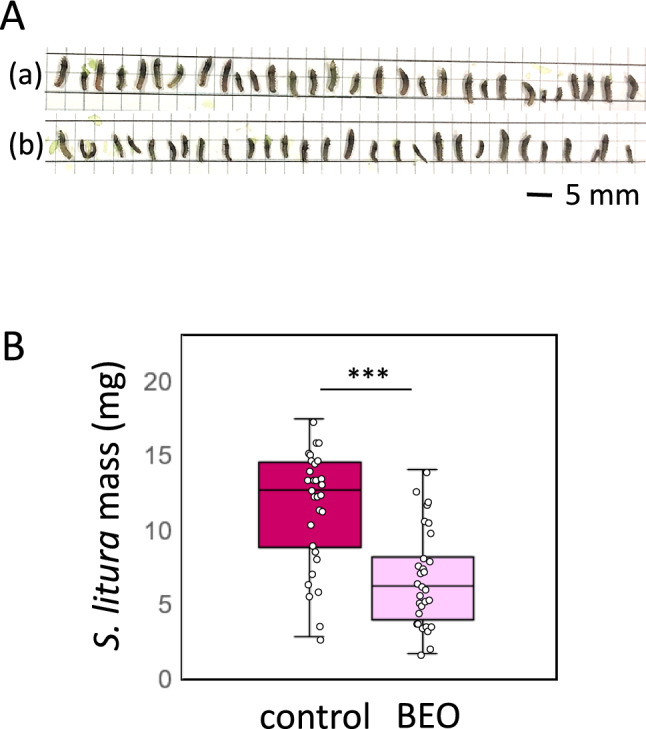
Effects of basil EO on the growth of *S. litura* larvae. **a** Second instar larvae of *S. litura* were fed (a) control or (b) basil EO-exposed tomato leaves. **b** Larval weights were determined at the end of the feeding trial. Bars represent means ± SDs. Significant differences were evaluated using Student’s *t* test (*P < 0.05; **P < 0.01; ***P < 0.001)

Next, we evaluated whether the higher expression of the wound response gene *Pin2* induced by basil EO in tomatoes could promote plant resistance to insect feeding. In this experiment, young *S. litura* larvae were fed tomato leaves, and changes in their growth were measured after the feeding period. The results showed that larvae-fed basil EO-exposed tomato leaves were smaller than larvae-fed control leaves (Fig. 10a). The weight of larvae-fed leaves pre-exposed to basil EO was approximately half of the control larvae weight (Fig. 10b).

## Discussion

Companion planting, which exploits compatibility between plant species to increase productivity per unit area and adaptability to environmental stresses, is expected to offer sustainable agriculture with reduced environmental impact. However, many studies have failed to produce results demonstrating these benefits, possibly due to the lack of scientific data supporting the effectiveness of companion plants, or the lack of well-established conditions and methods to detect their effectiveness. Clarification of the scientific basis for the benefits of companion plants is needed to establish effective strategies for their use in agricultural production.

In the present study, we established a mixed planting system consisting of tomato and basil plants to elucidate the molecular basis underlying the beneficial effects of companion plants on target plants. This experimental system showed that basil companion plants significantly enhanced the wounding response in tomato plants, which has previously been described as a priming effect (Mauch-Mani et al. 2017). We focused on plant–plant interactions through volatiles released from aboveground parts. Subsequently, we demonstrated that an EO prepared from basil leaves could prime the wounding response in tomato plants.

In plants, energy allocation to growth and stress responses typically follows a trade-off relationship, such that the induction of stress adaptation actively suppresses plant growth (Karasov et al. 2017). However, stress response priming induction has minimal effects on plant growth; it allows rapid and decisive responses to irregularly encountered stresses (Frost et al. 2008). Several molecular mechanisms are involved in the induction of plant-stress response priming (Pastora et al. 2013). Our experiments showed that basil volatiles induce MAPK expression and ROS production, both of which constitute essential mediators of plant-stress signaling (Meng and Zhang 2013). In Arabidopsis, benzothiadiazole activates plant-stress responses by inducing the expression of *AtMPK3* and *AtMPK6*, leading to enhanced expression of downstream disease resistance genes (Beckers et al. 2009). In addition, thiamine (i.e., vitamin B1) enhances the accumulation of ROS and callose during pathogen infection, resulting in H_2_O_2_-dependent induction of defense gene expression (Ahn et al. 2007). These chemicals may promote the accumulation of intracellular signaling factors and enhance downstream signaling (Pastora et al. 2013). The observed priming effect of basil volatiles, which enhanced the tomato wound response, is presumably driven by a similar mechanism. Basil volatiles promoted the expression of JA-related genes after wounding. Because MAPKs reportedly function as essential signal mediators in wound and JA-related responses (Seo et al. 2007; Takahashi et al. 2007), it is reasonable to speculate that basil volatiles activate or enhance MAPK-mediated JA signaling. Our findings suggest that ROS also function as critical mediators of volatile signaling. Several studies have demonstrated that ROS function both upstream and downstream of MAPKs (e.g., Jalmi and Sinha 2015).

We observed a similar priming effect in Arabidopsis exposed to basil EO. Loss-of-function analysis of Arabidopsis MAPKs strongly suggested that AtMPK3 and AtMPK6 are involved in basil EO-dependent defense priming. Although this effect was less pronounced than the effect observed in tomato plants, we detected a slight increase in ROS among wounded Arabidopsis leaves exposed to basil EO. This increase was not observed in *atmpk3* and *atmpk6* mutants, suggesting that MAPKs function upstream of ROS. We attempted to analyze the effects of basil EO on ROS accumulation in *atrborD:atrborF*, a double loss-of-function mutant of NADPH oxidoreductase; however, unfavorable growth conditions prevented us from completing the experiment. Further analyses of MAPK- and ROS-mediated pathways, including MAPK activation, are required. Although the involvement of other mechanisms for wound response priming has not been investigated, basil is expected to play a role in inducing this priming effect by amplifying intracellular signaling factors (e.g., MAPKs or ROS) in tomato plants through the release of volatiles.

The mechanism by which plants recognize volatiles as signals (i.e., their specific receptors) remains poorly understood. Thus far, ethylene is the only volatile compound that has been confirmed to act as a plant signal (Lacey and Binder 2014). However, beginning with studies of the poplar eavesdropping effect (Baldwin and Schultz 1983), various studies have revealed the potential for plant-derived volatile compounds to function as specific chemical signals. Recent studies have demonstrated that β-caryophyllene, released from insect-damaged plants, specifically binds to the transcriptional regulatory protein TOPLESS in tobacco cells and induces the expression of stress response-related genes (Nagashima et al. 2019). Stirling et al. (2024) have found that a petunia karrikin-insensitive receptor, PhKAI2ia, stereospecifically perceives ( −)-germacrene D to control its pistil development and seed yield. Airborne methyl salicylate is perceived and converted into salicylic acid by SABP2 in neighboring plants. Gong et al (2023) have revealed that methyl salicylate (MeSA), salicylic acid-binding protein-2 (SABP2), the transcription factor NAC2 and salicylic acid-carboxylmethyltransferase-1 (SAMT1) form a signaling circuit to mediate airborn defense against aphids and viruses. (Z)-3-Hexenol is another strong candidate for airborn defense to convert (Z)-3-hexenyl β-vicianoside in received tomato plants, and Sugimoto et al (2023) have identified that a uridine diphosphate-glycosyltransferase UGT91R1 catalyzes its production. SABP2 and UGT91R1 do not function as specific receptors but catalyze to generate other signaling molecules or valid substances in plant cells.

Intriguingly, plants may recognize the volatile signal as a blend of multiple compounds, rather than as a single compound (Kikuta et al. 2011). In the present study, we confirmed that four volatile compounds contained in basil EO play roles in the induction of wound response priming in tomato plants. In a future study, we will examine how different combinations of these four compounds influence wound responses in tomato and Arabidopsis plants. Our preliminary results indicate that mixed planting with basil substantially increases the symbiosis of mycorrhizal fungi in tomato plant roots (data not shown). Several studies have revealed that mycorrhizal fungi can prime disease resistance in plants (Pozo and Azcón-Aguilar 2007; Sabine et al. 2012). Interplant networks composed of mycorrhizal fungi mycelia are also suspected to function as communication tools in salicylic acid and JA signaling (Song et al. 2010, 2014). The ability of companion planting to enhance plant-stress adaptation through mycorrhizal fungi requires further study. Elucidation of the molecular origins of both above- and belowground interplant communication would substantially contribute to the global implementation of companion planting and future development of sustainable agriculture.

### Supplementary Information

Below is the link to the electronic supplementary material.

Supplementary file1 (DOCX 1028 KB)

## Data Availability

The datasets generated during and/or analyzed during the current study are available from the corresponding author on reasonable request.

## References

[CR1] Ahn I, Kim S, Lee Y, Suh S (2007) Vitamin B1-induced priming is dependent on hydrogen peroxide and the NPR1 gene in Arabidopsis. Plant Physiol 143:838–848. 10.1104/pp.106.092627 17158583 10.1104/pp.106.092627 PMC1803731

[CR2] Baldwin IT, Schultz JC (1983) Rapid changes in tree leaf chemistry induced by damage: evidence for communication between plants. Science 221:277–279. 10.1126/science.221.4607.277 17815197 10.1126/science.221.4607.277

[CR3] Beckers GJM, Jaskiewicz M, Liu Y et al (2009) Mitogen-activated protein kinases 3 and 6 are required for full priming of stress responses in *Arabidopsis thaliana*. Plant Cell 21:944–953. 10.1105/tpc.108.062158 19318610 10.1105/tpc.108.062158 PMC2671697

[CR4] Ben-Issa R, Gomez L, Gautier H (2017a) Companion plants for aphid pest management. InSects 8:112. 10.3390/insects8040112 29053585 10.3390/insects8040112 PMC5746795

[CR5] Ben-Issa R, Gautier H, Gomez L (2017b) Influence of neighbouring companion plants on the performance of aphid populations on sweet pepper plants under greenhouse conditions. Agric for Entomol 19:181–191. 10.3390/insects8040112 10.3390/insects8040112

[CR6] Bomford MK (2004) Yield, pest density, and tomato flavor effects of companion planting in garden-scale studies incorporating tomato, basil, and brussels sprout (Morgantown, WV: West Virginia University). 10.33915/etd.2105

[CR7] Boter M, Ruíz-Rivero O, Abdeen A et al (2004) Conserved MYC transcription factors play a key role in jasmonate signaling both in tomato and Arabidopsis. Genes Dev 18(13):1577–1591. 10.1101/gad.297704 15231736 10.1101/gad.297704 PMC443520

[CR8] Conboy NJA, McDaniel T, Ormerod A et al (2019) Companion planting with French marigolds protects tomato plants from glasshouse whiteflies through the emission of airborne limonene. PLoS ONE 14:e0213071. 10.1371/journal.pone.0213071 30822326 10.1371/journal.pone.0213071 PMC6396911

[CR9] Conrath U (2011) Molecular aspects of defense priming. Trends Plant Sci 16(10):524–531. 10.1016/j.tplants.2011.06.004 21782492 10.1016/j.tplants.2011.06.004

[CR10] Du M, Zhao J, Tzeng DTW, Liu Y et al (2014) MYC2 orchestrates a hierarchical transcriptional cascade that regulates jasmonate-mediated plant immunity in tomato. Plant Cell 29(8):1883–1906. 10.1105/tpc.16.00953 10.1105/tpc.16.00953 PMC5590496 28733419

[CR11] Engelberth J, Alborn HT, Schmelz EA et al (2004) Airborne signals prime plants against insect herbivore attack. Proc Natl Acad Sci U S A 101(6):1781–1785. 10.1073/pnas.0308037100 14749516 10.1073/pnas.0308037100 PMC341853

[CR12] Farmer EE, Ryan CA (1992) Octadecanoid precursors of jasmonic acid activate the synthesis of wound-inducible proteinase inhibitors. Plant Cell 4(2):129–134. 10.1105/tpc.4.2.129 12297644 10.1105/tpc.4.2.129 PMC160114

[CR13] Finch S, Billiald H, Collier R (2003) Companion planting–do aromatic plants disrupt host-plant finding by the cabbage root fly and the onion fly more effectively than non-aromatic plants? Entomol Experimentalis Applicata 109(3):183–195. 10.1007/978-981-10-4325-3_10 10.1007/978-981-10-4325-3_10

[CR14] Frost CJ, Mescher MC, Carlson JE et al (2008) Plant defense priming against herbivores: getting ready for a different battle. Plant Physiol 146:818–824. 10.1104/pp.107.113027 18316635 10.1104/pp.107.113027 PMC2259053

[CR15] Fu X, Wu X, Zhou X et al (2015) Companion cropping with potato onion enhances the disease resistance of tomato against *Verticillium dahliae*. Front Plant Sci 11(6):726. 10.3389/fpls.2015.00726 10.3389/fpls.2015.00726 PMC4566073 26442040

[CR16] Gao H, Tian H, Zhang Z et al (2022) Warming-induced greenhouse gas fluxes from global croplands modified by agricultural practices: a meta-analysis. Sci Total Environ 820:153288. 10.1016/j.scitotenv.2022.153288 35066045 10.1016/j.scitotenv.2022.153288

[CR17] George DR, Collier RH, Whitehouse DM (2013) Can imitation companion planting interfere with host selection by Brassica pest insects? Agric for Entomol 15(1):106–109. 10.1111/j.1461-9563.2012.00598.x 10.1111/j.1461-9563.2012.00598.x

[CR18] Giller KE, Hijbeek R, Andersson JA et al (2021) Regenerative agriculture: an agronomic perspective. Outlook Agri 50(1):13–25. 10.1177/0030727021998063 10.1177/0030727021998063 PMC8023280 33867585

[CR19] Gong Q, Wang Y, He L et al (2023) Molecular basis of methyl-salicylate-mediated plant airborne defence. Nature 622(7981):139–148. 10.1038/s41586-023-06533-3 37704724 10.1038/s41586-023-06533-3

[CR20] Higgins R, Lockwood T, Holley S et al (2007) Changes in extracellular pH are neither required nor sufficient for activation of mitogen-activated protein kinases (MAPKs) in response to systemin and fusicoccin in tomato. Planta 225(6):1535–1546. 10.1007/s00425-006-0440-8 17109147 10.1007/s00425-006-0440-8

[CR21] Horrigan L, Lawrence RS, Walker P (2002) How sustainable agriculture can address the environmental and human health harms of industrial agriculture. Environ Health Perspect 110(5):445–456 12003747 10.1289/ehp.02110445 PMC1240832

[CR22] Jalmi SK, Sinha AK (2015) ROS mediated MAPK signaling in abiotic and biotic stress-striking similarities and differences. Front Plant Sci 6:769. 10.3389/fpls.2015.00769 26442079 10.3389/fpls.2015.00769 PMC4585162

[CR23] Kandoth PK, Ranf S, Pancholi SS et al (2009) Tomato MAPKs LeMPK1, LeMPK2, and LeMPK3 function in the systemin-mediated defense response against herbivorous insects. Proc Natl Acad Sci USA 104(29):12205–12210. 10.1073/pnas.0700344104 10.1073/pnas.0700344104 PMC1924534 17623784

[CR24] Karasov TL, Chae E, Herman JJ et al (2017) Mechanisms to mitigate the trade-off between growth and defense. Plant Cell 29(4):666–680. 10.1105/tpc.16.00931 28320784 10.1105/tpc.16.00931 PMC5435432

[CR25] Kikuta Y, Ueda H, Nakayama K et al (2011) Specific regulation of pyrethrin biosynthesis in *Chrysanthemum cinerariaefolium* by a blend of volatiles emitted from artificially damaged conspecific plants. Plant Cell Physiol 52(3):588–596. 10.1093/pcp/pcr017 21296762 10.1093/pcp/pcr017

[CR26] Lacey RF, Binder BM (2014) How plants sense ethylene gas–the ethylene receptors. J Inorg Biochem 133:58–62. 10.1016/j.jinorgbio.2014.01.006 24485009 10.1016/j.jinorgbio.2014.01.006

[CR27] Li L, Zhao Y, McCaig BC et al (2003) The tomato homolog of CORONATINE-INSENSITIVE1 is required for the maternal control of seed maturation, jasmonate-signaled defense responses, and glandular trichome development. Plant Cell 16:126–143 14688297 10.1105/tpc.017954 PMC301400

[CR28] Mauch-Mani B, Baccelli I, Luna E et al (2017) Defense priming: an adaptive part of induced resistance. Annu Rev Plant Biol 68:485–512. 10.1146/annurev-arplant-042916-041132 28226238 10.1146/annurev-arplant-042916-041132

[CR53] Meng X, Zhang S (2013) MAPK cascades in plant disease resistance signaling. Ann Rev Phytopathol 51(1):245-266. 10.1146/annurev-phyto-082712-102314 23663002 10.1146/annurev-phyto-082712-102314

[CR29] Mengel K (2001) Alternative or complementary role of foliar supply in mineral nutrition. International Symposium on Foliar Nutrition of Perennial Fruit Plants. Acta Sci Pol-Hortoru 594:33–47

[CR30] Moss B (2007) Water pollution by agriculture. Philos Trans R Soc Lond B Biol Sci 363(1491):659–666 10.1098/rstb.2007.2176 PMC2610176 17666391

[CR31] Nagashima A, Higaki T, Koeduka T et al (2019) Transcriptional regulators involved in responses to volatile organic compounds in plants. J Biol Chem 294(7):2256–2266. 10.1074/jbc.ra118.005843 30593507 10.1074/jbc.ra118.005843 PMC6378981

[CR32] Parker JE, Snyder WE, Hamilton GC et al (2013) Companion planting and insect pest control Weed and Pest Control-Conventional and New Challenges (IntechOpen)10.5772/55044

[CR33] Pastora V, Lunab E, Mauch-Manic B et al (2013) Primed plants do not forget. Environ Exp Bot 94:46–56. 10.1016/j.envexpbot.2012.02.013 10.1016/j.envexpbot.2012.02.013

[CR34] Pickett JA, Woodcock CM, Midega CAO et al (2014) Push–pull farming systems. Curr Opin Biotechnol 26:125–132. 10.1016/j.copbio.2013.12.006 24445079 10.1016/j.copbio.2013.12.006

[CR35] Pleasant JM (2016) Food yields and nutrient analyses of the three sisters: a haudenosaunee cropping system. Ethnobiol Lett 7:87–98

[CR36] Politeo O, Jukic M, Milos M (2007) Chemical composition and antioxidant capacity of free volatile aglycones from basil (*Ocimum basilicum* L.) compared with its essential oil. Food Chem 101:379–385. 10.1016/j.foodchem.2006.01.045 10.1016/j.foodchem.2006.01.045

[CR37] Postor V, Luna E et al (2013) Fine tuning of reactive oxygen species homeostasis regulates primed immune responses in Arabidopsis. MPMI 26:1334–1344. 10.1094/MPMI-04-13-0117-R 24088017 10.1094/MPMI-04-13-0117-R

[CR38] Pozo MJ, Azcón-Aguilar C (2007) Unraveling mycorrhiza-induced resistance. Curr Opin Plant Biol 10(4):393–398. 10.1016/j.pbi.2007.05.004 17658291 10.1016/j.pbi.2007.05.004

[CR39] Ryan CA, Pearce G (1998) Systemin: a polypeptide signal for plant defensive genes. Annu Rev Cell Dev Biol 14:1–17. 10.1146/annurev.cellbio.14.1.1 9891776 10.1146/annurev.cellbio.14.1.1

[CR40] Sabine C, Martinez-Medina JA, Lopez-Raez JA et al (2012) Mycorrhiza-induced resistance and priming of plant defenses. J Chem Ecol 38:651–664. 10.1007/s10886-012-0134-6 22623151 10.1007/s10886-012-0134-6

[CR41] Seo S, Katou S, Seto H et al (2007) The mitogen-activated protein kinases WIPK and SIPK regulate the levels of jasmonic and salicylic acids in wounded tobacco plants. Plant J 49(5):899–909. 10.1111/j.1365-313x.2006.03003.x 17253983 10.1111/j.1365-313x.2006.03003.x

[CR42] Song YY, Zeng RS, Xu JF et al (2010) Interplant communication of tomato plants through underground common mycorrhizal networks. PLoS ONE 5(10):e13324. 10.1371/journal.pone.0013324 20967206 10.1371/journal.pone.0013324 PMC2954164

[CR43] Song YY, Ye M, Li C et al (2014) Hijacking common mycorrhizal networks for herbivore-induced defence signal transfer between tomato plants. Sci Rep 4:3915. 10.1038/srep03915 24468912 10.1038/srep03915 PMC3904153

[CR44] Song LX, Xu XC, Wang FN et al (2018) Brassinosteroids act as a positive regulator for resistance against root-knot nematode involving RESPIRATORY BURST OXIDASE HOMOLOG-dependent activation of MAPKs in tomato. Plant Cell Environ 41(5):1113–1125. 10.1111/pce.12952 28370079 10.1111/pce.12952

[CR45] Stirling SA, Guercio AM, Patrick RM et al (2024) Volatile communication in plants relies on a KAI2-mediated signaling pathway. Science 383(6689):1318–1325. 10.1126/science.adl4685 38513014 10.1126/science.adl4685

[CR46] Stirling SA, Guercio AM, Patrick RM et al (2024) Volatile communication in plants relies on a KAI2-mediated signaling pathway. Science 383(6689):1318–1325 38513014 10.1126/science.adl4685

[CR47] Sugimoto K, Ono E, Inaba T et al (2023) Identification of a tomato UDP-arabinosyltransferase for airborne volatile reception. Nat Commun 14(1):677. 10.1038/s41467-023-36381-8 36755045 10.1038/s41467-023-36381-8 PMC9908901

[CR48] Stulemeijer IJE, Stratmann JW, Joosten MHAJ (2009) Tomato mitogen-activated protein kinases LeMPK1, LeMPK2, and LeMPK3 are activated during the Cf-4/Avr4-induced hypersensitive response and have distinct phosphorylation specificities. Plant Physiol 144(3):1481–1494. 10.1104/pp.107.101063 10.1104/pp.107.101063 PMC1914120 17478632

[CR49] Sukegawa S, Shiojiri K, Higami T et al (2018) Pest management using mint volatiles to elicit resistance in soy: mechanism and application potential. Plant J 96:910–920. 10.1111/tpj.14077 30156351 10.1111/tpj.14077

[CR50] Takahashi F, Yoshida R, Ichimura K et al (2007) The mitogen-activated protein kinase cascade MKK3-MPK6 is an important part of the jasmonate signal transduction pathway in Arabidopsis. Plant Cell 19(3):805–818. 10.1105/tpc.106.046581 17369371 10.1105/tpc.106.046581 PMC1867372

[CR51] Takahashi Y, Shiojiri K, Yamawo A (2021) Aboveground plant-to-plant communication reduces root nodule symbiosis and soil nutrient concentrations. Sci Rep 11:12675. 10.1038/s41598-021-92123-0 34135405 10.1038/s41598-021-92123-0 PMC8209107

[CR52] Tongnuanchan P, Benjakul S (2014) Essential oils: extraction, bioactivities, and their uses for food preservation. J Food Sci 79:1231–1249. 10.1111/1750-3841.12492 10.1111/1750-3841.12492 24888440

