# Supplementary material for: Companion basil plants prime the tomato wound response through volatile signaling in a mixed planting system
Source: Plant Cell Rep. 2024 Jul 22;43(8):200. doi: 10.1007/s00299-024-03285-w (PMC11263239; doi:10.1007/s00299-024-03285-w)
Supplement: Supplementary file 1 — Supplementary file1 (DOCX 1028 KB) [file 299_2024_3285_MOESM1_ESM.docx]

**Supplementary Information**

**Companion basil plants prime the tomato wound response through volatile signaling in a mixed planting system**

Plant Cell Reports

**Authors:** Riichiro Yoshida^1,2^, Shoma Taguchi^1^, Chihiro Wakita^1^, Shinichiro Serikawa^1^, Hiroyuki Miyaji^1^

**Affiliations:**

^1^Laboratory of Horticultural Science, Faculty of Agriculture, Kagoshima University, 1-21-24 Kohrimoto, Kagoshima, Kagoshima 890-0065

^2^The United Graduate School of Agricultural Sciences, Kagoshima University, 1-21-24 Kohrimoto, Kagoshima, Kagoshima, 890-0065, Japan

Correspondence to: ryoshida@agri.kagoshima-u.ac.jp

Supplementary Table

Table S1. Information of primers and gene used in this study.

| Genes name | Forward primer | Reverse primer |
| --- | --- | --- |
| *Pin2* | GGATTTAGCGGACTTCCTTCTG | ATGCCAAGGCTTGTACTAGAGAATG |
| *LOXD* | ATCTCCCAAGTGAAACACCACA | TCATAAACCCTGTCCCATTCTTC |
| *AOS* | TACCAACCATTTGCAACGAA | CGCACTGTTTATTCCCCACT |
| *AOC* | CTCGGAGATCTTGTCCCCTTT | CTCCTTTCTTCTCTTCTTCGTGCT |
| *PSYS* | TCACCATGAGAAGGGAGGAG | TGCATCATCATCTCCTTCAA |
| *MYC2* | AGCAGGAGCATCGGAAGAA | CCAAATCGGGCTGGAACTA |
| *SIMPK1* | GGGCTAGCTCGTGTCACTTC | GGAGTGCCAATCAACTCCAT |
| *SIMPK2* | AATTCCACCACCTCAACGAG | GGCTTCAAGTCCCTATGCAA |
| *SIMPK3* | CCGGAGCTTTTGTTGAACTC | GTGGGAGTTGCCTGACGTAT |
| *Wfi1* | CTGCTTGGAAGAAGAAATC | GAATTTTGCATCGTACAATAG |
| *VSP2* | TCAGTGACCGTTGGAAGTTGTG | GTTCGAACCATTAGGCTTCAATATG |
| *Tomato ACTIN* | AGACGCCTATGTGCGGAGATG | GAGGACAGGATGCTCCTCAG |
| *Arabidopsis ACTIN* | AGTGGTCGTACAACCGGTATTGT | GATGGCATGAGGAAGAGAGAAAC |

Supplementary Figures


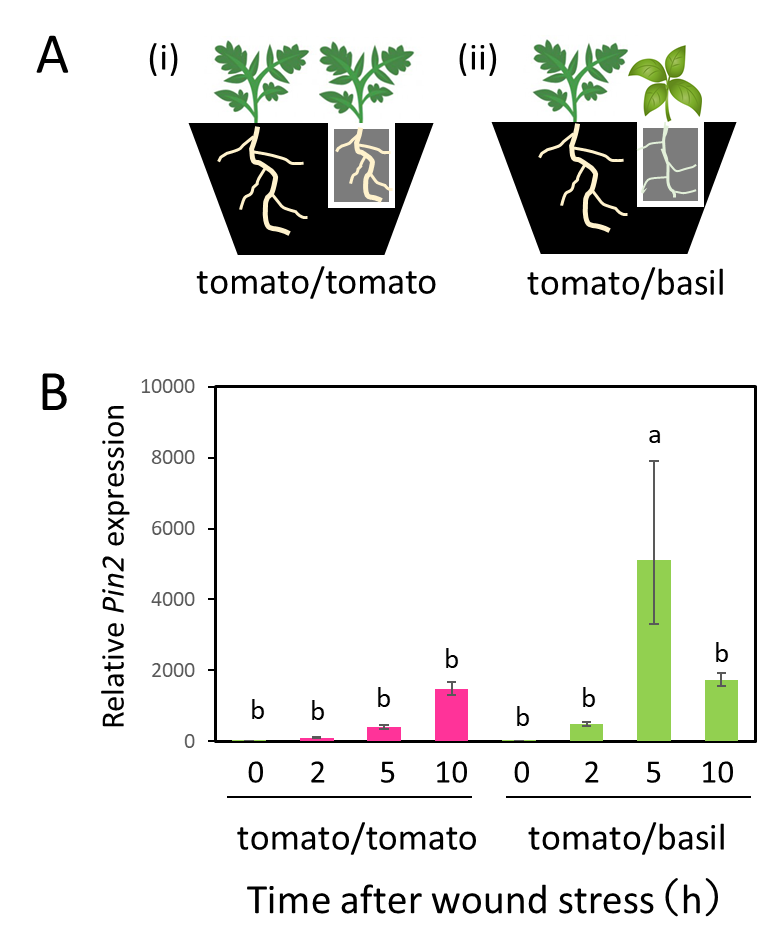


**Figure S1.** Experiment to evaluate above- and belowground priming of the tomato wound response by basil plants. (A) Two experimental plots were established: (i) tomato plants without basil; (ii) mixed planting of tomato and basil plants, with a separate small plant box without holes (3-cm-diameter) growing basil plant to avoid any contact between their roots during the growth period. (B) Effects of basil on expression of the wound response gene *Pin2* in tomato leaves. Leaves were wounded on both sides with scissors, sampled at the indicated times, and subjected to qPCR analysis. Bars represent means ± SDs from three independent experiments. Different letters indicate significant differences (*P* < 0.05, one-way ANOVA followed by Tukey’s test; n = 3).

**
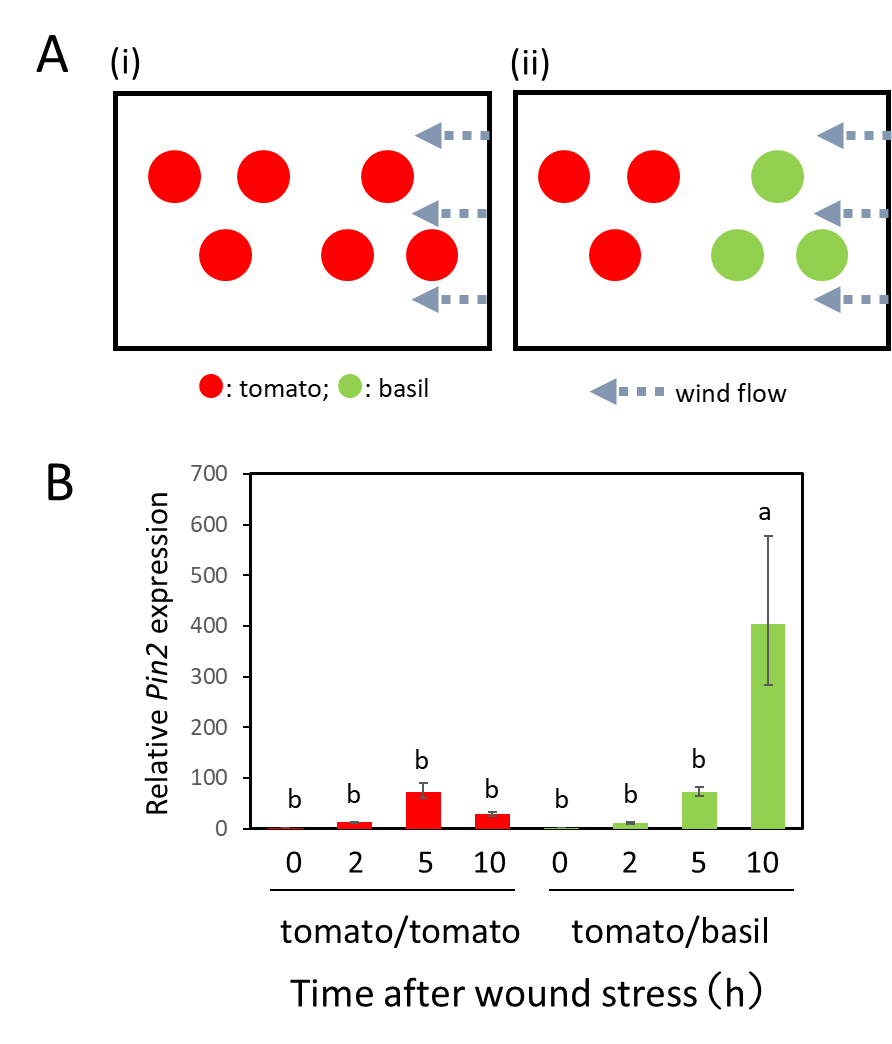
**

**Figure S2.** Experiment to evaluate above- and belowground priming of the tomato wound response by basil plants. (A) Two experimental plots were established: (i) six tomato pots were placed in a container (54.4 cm length × 26.2 cm width × 30.5 cm height); (ii) three tomato and three basil pots were placed in a container. The containers were set up to allow a moderate breeze to flow from the basil side to the tomato side. (B) Effects of basil on expression of the wound response gene *Pin2* in tomato leaves. Leaves were wounded on both sides with scissors, sampled at the indicated times, and subjected to qPCR analysis. Bars represent means ± SDs from three independent experiments. Different letters indicate significant differences (*P* < 0.05, one-way ANOVA followed by Tukey’s test; n = 3).


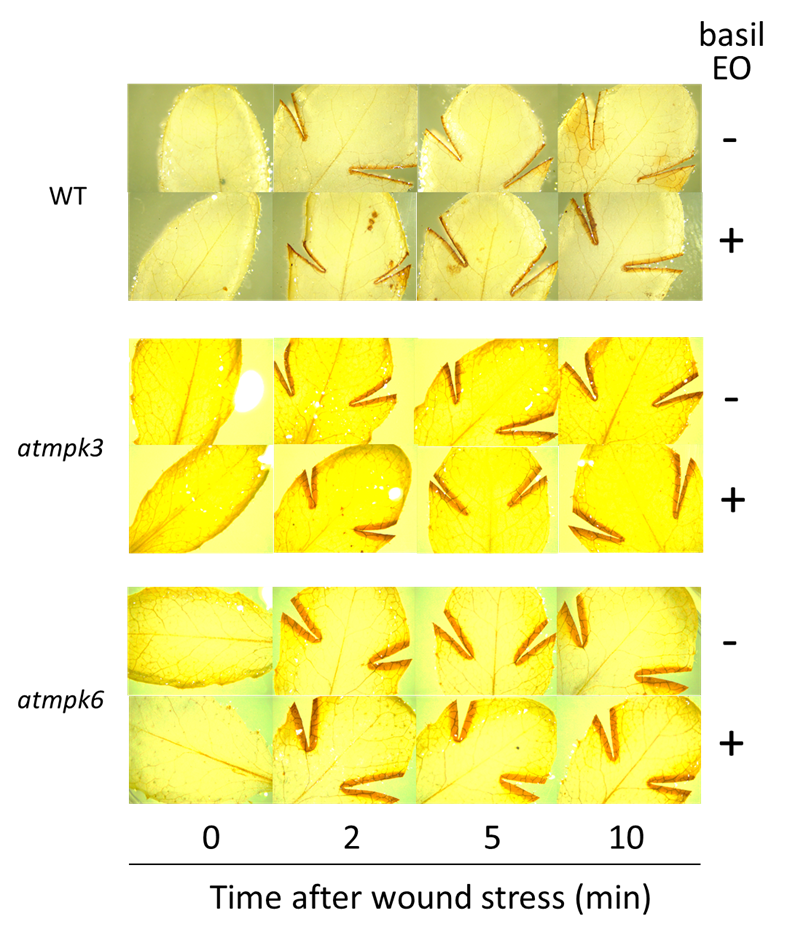


**Fig. S3** DAB staining of wounded Arabidopsis leaves. WT, *atmpk3*, and *atmpk6* plants were pre-exposed to basil EO for 15 h, then wounded with scissors. – and + indicate the absence and presence, respectively, of pre-exposure to basil EO.
